# Supplementary material for: High genomic plasticity and unique features of Xanthomonas translucens pv. graminis revealed through comparative analysis of complete genome sequences
Source: BMC Genomics. 2023 Dec 5;24:741. doi: 10.1186/s12864-023-09855-8 (PMC10699075; doi:10.1186/s12864-023-09855-8)
Supplement: Supplementary file 1 — Supplementary Material 1 [file 12864_2023_9855_MOESM1_ESM.docx]

**Table S1. *Xanthomonas translucens* strains included in the gene content analysis and characteristics of the genome assemblies.**

| Clade | Pathovar | Strain | Country | Isolated from | Chromosome length (bp) | Plasmid length (bp) | Accession | Reference |
| --- | --- | --- | --- | --- | --- | --- | --- | --- |
| Xt-I | *translucens* | B1FA | USA | *Hordeum vulgare* | 4,661,643 | NA | CP090000 | (42) |
|  | *translucens* | B8GF | USA | *Hordeum vulgare* | 4,708,077 | NA | CP089999 | (42) |
|  | *translucens* | CIX43 | USA | *Hordeum vulgare* | 4,664,501 | 36,413 | CP072988-CP072989 | (43) |
|  | *translucens* | CIX95 | USA | *Hordeum vulgare* | 4,647,206 | NA | CP072990 | (43) |
|  | *translucens* | XtKm7 | Iran | *Hordeum vulgare* | 4,577,861 | NA | CP064005 | (44) |
|  | *translucens* | XtKm8 | Iran | *Hordeum vulgare* | 4,792,950 | NA | CP064004 | (44) |
|  | *translucens* | XtKm9 | Iran | *Hordeum vulgare* | 4,689,955 | NA | CP064003 | (44) |
|  | *translucens* | XtKm34 | Iran | *Hordeum vulgare* | 4,680,513 | NA | CP064001 | (44) |
|  | *hordei* | UPB458 | India | *Hordeum vulgare* | 4,679,124 | NA | CP076249 | (3) |
|  | *undulosa* | Xtu 4699 | USA | *Triticum spp.* | 4,561,137 | NA | CP008714 | (18) |
|  | *undulosa* | ICMP 11055 | Iran | *Triticum spp.* | 4,761,583 | NA | CP009750 | (45) |
|  | *undulosa* | LW16 | USA | *Triticum spp.* | 4,746,074 | NA | CP043540 | (18) |
|  | *undulosa* | P3 | USA | *Triticum spp.* | 4,618,583 | NA | CP043500 | (18) |
|  | *undulosa* | XtFa1 | Iran | *Triticum spp.* | 4,605,208 | NA | CP063996 | (44) |
|  | *undulosa* | XtKm12 | Iran | *Triticum spp.* | 4,581,137 | NA | CP064000 | (44) |
|  | *undulosa* | XtKm15 | Iran | *Lolium perenne* | 4,560,646 | 41,956 45,639 | CP063997-CP063999 | (44) |
|  | *undulosa* | XtKm33 | Iran | *Hordeum vulgare* | 4,626,215 | NA | CP064002 | (44) |
|  | *undulosa* | XtLr8 | Iran | *Triticum spp.* | 4,563,212 | 45,351 40,770 | CP063993-CP063995 | (44) |
|  | *undulosa* | CFBP 2055 | Canada | *Triticum spp.* | 4,607,252 | 46,036 | CP074361-CP074362 | (3) |
|  | *undulosa* | MAI5034 | Uruguay | *Triticum spp.* | 4,625,916 | NA | CP089584 | (46) |
|  | *undulosa* | UPB513 | Mexico | *Triticum spp.* | 4,613,657 | 90,481 | CP096570-CP096569 | Toth et al, unpublished |
|  | *secalis* | CFBP 2539 | Canada | *Secale cereale* | 4,565,955 | NA | CP074363 | (3) |
|  | *pistaciae* group A | CFBP 8304 | Australia | *Pistacia vera* | 4,599,174 | NA | CP074365 | (3) |
| Xt-II | *cerealis* | NXtc01 | China | *Triticum spp.* | 4,622,298 | NA | CP038228 | (47) |
|  | *pistaciae* group B | ICMP 16317 | Australia | *Pistacia vera* | 4,386,175 | NA | CP083804 | (3) |
