# Supplementary figures and images for: High genomic plasticity and unique features of Xanthomonas translucens pv. graminis revealed through comparative analysis of complete genome sequences
Source: BMC Genomics. 2023 Dec 5;24:741. doi: 10.1186/s12864-023-09855-8 (PMC10699075; doi:10.1186/s12864-023-09855-8)

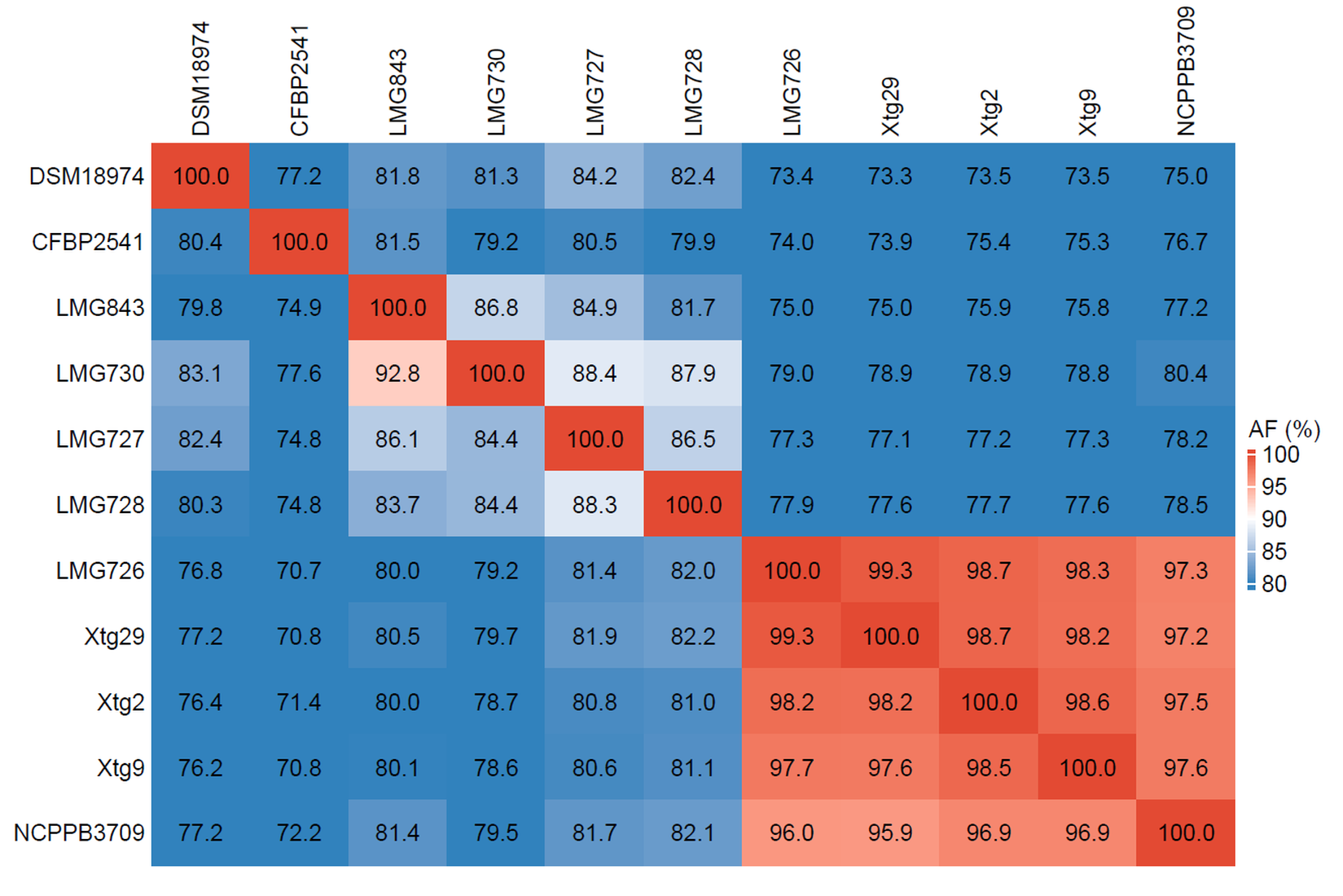


**Figure S1** Alignment fraction (AF) used in each comparison for the ANI calculations

Supplement: Supplementary file 3 — Supplementary Material 3 [file 12864_2023_9855_MOESM3_ESM.docx]
